# Prevalence and associated risk factors of bovine *Schistosoma* and *Fasciola* infections among cattle in South Achefer District, North West Ethiopia

**DOI:** 10.1016/j.parepi.2025.e00415

**Published:** 2025-01-30

**Authors:** Solomon Tesfaye, Mastewal Yeshambel, Amir Alelign, Tilahun Yohannes

**Affiliations:** Department of Biology, College of Natural and Computational Science, University of Gondar, Gondar, Ethiopia

**Keywords:** Bovine fasciolosis, Bovine schistosomiasis, Prevalence, Risk factors, South Achefer

## Abstract

Bovine schistosomiasis and fasciolosis, caused by flatworms from different species of *Schistosoma* and *Fasciola*, continue to be significant animal health concerns in cattle farming in developing countries like Ethiopia. However, not enough epidemiological information is available in most parts of Ethiopia in this respect. Therefore, the present study aimed to determine the magnitude of these two important zoonotic diseases and the associated risk factors in south Achefer district, northwest Ethiopia. A cross-sectional study was carried out in June and August of 2020. The research cattle, which belonged to farmers in three selected localities, were chosen by a simple random sampling method. Freshly collected faecal samples were subjected to a parasitological test using the sedimentation technique to identify eggs of *Schistosoma* and *Fasciola*. We used Pearson's chi-square (χ2) test to ascertain the degree of variation between the proportions of disease occurrence. Logistic regression analyses were performed for the risk factor analysis. The overall prevalence of *Schistosoma bovis* and *Fasciola hepatica* was 9.6 % (37/384) and 54.2 % (208/384), respectively. Cross-bred cattle had around twice the odds of contracting *Fasciola hepatica* than local cattle (adjusted odds ratio, AOR: 1.87, 95 % CI: 1.02–3.43). The prevalence of *Fasciola hepatica* was more than seven times higher in younger cattle than in older cattle (AOR: 7.31, 95 % CI: 3.54–15.08). Comparatively to cattle in good physical health, those in poor physical condition were shown to contract *Fasciola hepatica* more than four times (AOR: 4.85, 95 % CI: 1.94–12.14). This study indicated that bovine *Schistosoma* and *Fasciola* infections remain among the major cattle health problems in the study area. Therefore, appropriate intervention methods should be implemented for effective zoonotic disease control in the study area.

## 1 Introduction

Fasciolosis and schistosomiasis are two significant threats to cattle health worldwide (Okpala et al., 2004; Ahmad et al., 2021). Worms from the genera *Schistosoma* and *Fasciola* cause these zoonotic illnesses (Mas-Coma et al., 2018; McManus et al., 2018). The spread of these infections is heavily influenced by the ecology of the aquatic snail intermediate hosts (Asrese and Ali, 2014: Lu et al., 2018). The livestock industry in Ethiopia and other parts of Africa is abundant, but it doesn't reach its full potential due to diseases like fasciolosis and schistosomiasis, as well as related issues such as inadequate management practices, ineffective livestock development services, and low genetic potential in local breeds (Demlew and Tessma, 2020; Duguma, 2020).

The disease schistosomiasis, also known as bilharziasis, is caused by several species of the genus *Schistosoma* and is widespread in many tropical and subtropical regions of Africa and Asia. Species such as *Schistosoma bovis* (*S. bovis), S. mathew*, *S. intercalatum*, *S. spindale*, *S. nasalis*, and *S. indicum* have significant veterinary importance for livestock production in endemic areas around the world. *Schistosoma bovis* is the most frequent species in Africa and the Mediterranean, while in Asia, schistosomiasis is primarily caused by *S. spindale*, *S. indicum*, and *S. nasalis.* The spread of the disease is mainly influenced by the location of snail intermediate hosts, particularly the *Bulinus* and *Physopsis* species, which are significant for bovine schistosomiasis (Lu et al., 2018).

Bovine fasciolosis is a significant parasite illness that affects cattle in different regions of Ethiopia. It leads to both mortality and reduced productivity. The disease is endemic in both highlands and lowlands areas. It is caused by two trematodes, *Fasciola hepatica* (*F. hepatica)* (the common liver fluke) and *Fasciola gigantica* (*F. gigantica*). Bovine fasciolosis is a plant-borne trematode zoonosis and is classified as a neglected tropical disease (NTD). Although it affects humans, its main host is ruminants such as cattle and sheep. In Europe, America, and Oceania, only *F. hepatica* is of concern, but both species overlap in many areas of Africa and Asia (Mas-Coma et al., 2005).

Schistosomiasis and fasciolosis can lead to significant economic losses, reduced productivity, low milk yield, and increased susceptibility to other diseases in livestock (Asrese and Ali, 2014; Zewde et al., 2019; Demlew and Tessma, 2020). The main transmission locations include small streams across Ethiopia's highlands, lakes such as Tana and Zeway, irrigation systems in Qoga Dam, and the sugar state in Wongi (Alemu, 2019; Yihunie et al., 2019).

Our observations showed that the research area is characterized by wetlands that provide a habitat for biological vectors of schistosomiasis and fasciolosis. However, the area faces challenges such as inadequate management and low literacy among farmers regarding fasciolosis and bovine schistosomiasis. This is due to the lack of information to help with the management of cattle and the prevention and control of these diseases in the study area. To address this, the study was carried out to gather essential data and bridge the knowledge gap about the prevalence of bovine schistosomiasis and fasciolosis in the South Achefer district of northwest Ethiopia.

## 2 Materials and methods

### 2.1 Study area and duration

The study was conducted in the South Achefer District of the Amhara Region, northwest Ethiopia, between June 2020 and August 2020. The district is located about 503 km northwest of Addis Ababa, the capital city of Ethiopia, and about 65 km southwest of Bahir Dar, the capital city of the region. The altitude of the district ranges from 1800 to 2800 m above sea level, and it covers a total surface area of 159,027 ha. The district lies between the coordinates of 11^o^51’N latitude and 37^o^10’E longitude. According to the Amhara National Regional State Agricultural Office, the annual rainfalls is 1703 mm, and the minimum, maximum, and mean annual temperatures are 5.70 °C, 30.60 °C, and 18.80 °C, respectively (ANRSAO (Amara National Regional State Agricultural and Development Office), 2011).

The district comprises 20,106 equines, 205,767 cattle, 148,971 ovine (sheep), and 204,181 poultry. The landscape of the study area is marked by its location near the Blue Nile River with Ashare and Kiltei irrigation. Livestock is reared in the area with mixed farming; thus, farm animals are the major source of agricultural income for livestock owners (ANRSAO (Amara National Regional State Agricultural and Development Office), 2011). Currently, the district consists of 25 peasant associations (PAs.) and four urban Kebeles (a small administrative unit in Ethiopia), and the rest, 21 Kebeles, are in rural areas. Out of 25 PAs, three of them are found in the ‘Woinadega’ agroecological zone, which is characterized by marshy stagnant water bodies, a large number of irrigation canals, and rivers near the study area.

The study focused on three PAs: Abchikli Zuriya, Yeboden Gergista, and Ker Gurach. These PAs operate a large free-grazing husbandry system near the Blue Nile River. Farmers in the study area practiced extensive, semi-intensive, and intensive livestock farming systems. In the extensive system, animals were often kept outdoors and grazed freely near large swampy fields such as Ashar and Chometmesk, with a small irrigation dam in the Blue Nile River. These grazing areas posed a potential risk for *Schistosoma* infection due to animals' frequent contact with the water. In the semi-intensive management system, cattle were kept partly indoors and outdoors, with indoor supplementation of quality food and clean water.

### 2.2 The study design and population

A community-based cross-sectional study was carried out in three localities. The study included a total of 384 cattle of varying breeds, ages, sexes, body conditions, origins, and management systems. Body conditions were categorized as poor, medium, and good based on (Ferguson et al., 1994).

### 2.3 Inclusion and exclusion criteria

The study included both local and cross-bred cattle that were older than six months. Cows that were one month pre- and post-partum, clinically unwell cattle and young cattle younger than six months were excluded from the study. Additionally, cattle that had been administered the drug praziquantel before the start of the study was also excluded.

### 2.4 Sample size determination and sampling technique

The sample size (n) was calculated using the single population proportion formula for a cross-sectional study (Woodward, 2013), based on the following assumptions: a 5 % margin of error, 95 % confidence interval, and a 51.3 % prevalence of *Fasciola hepatica* (Mohammed et al., 2018). This resulted in a sample size of 384 cattle. We utilized purposive sampling to select three rural Kebeles in the study area and then employed simple random sampling to choose the 384 cattle from the selected Kebeles.

### 2.5 Data collection procedures

#### 2.5.1 Parasitological study

Faecal samples were collected directly from the cattle rectum using sterile gloves. The collected samples were placed in a clean universal bottle, preserved with 10 % formalin to prevent the hatching of miracidia, and closed with a screw top in an airtight condition until the parasitological examination.

The samples were all clearly label1ed with important information such as the sampling date, sex, breed type, age, and body condition score of each cattle. Additionally, the selected peasant associations and cattle management systems used by the peasants were also recorded. The collected samples were examined qualitatively using the sedimentation technique described by Hansen and Perry (1994). To explain briefly, approximately 5 g of faecal sample were mixed with 40 ml of tap water in a centrifuge tube and thoroughly mixed. The faecal suspension was then filtered through a tea strainer into a beaker. The filtered material was transferred into a test tube and allowed to sit for 20 to 25 min. The supernatant fluid was carefully discarded, and a drop of sediment was transferred to a slide using a pipette. The slide was then stained with 1 % methylene blue and examined under a microscope for the presence of parasite eggs. The slides were considered positive for *Schistosoma bovis* if they showed oval or spindle-shaped eggs, which are characterized by a central bulge and a terminal spine on one side. In contrast, *Fasciola hepatica* eggs were identified by their distinct yellowish color, larger size, and thin operculated shells (Elsheikha and Khan, 2011).

### 2.6 Data analysis method

The data were properly coded and thoroughly inspected for errors before any statistical analyses were performed. We utilized the Statistical Package for the Social Sciences (SPSS) version 21.0 (SPSS Inc., Chicago, IL, USA) to analyze the data after importing it from Microsoft Excel. Descriptive statistics were employed to determine the frequency of fascioliasis and schistosomiasis. To assess the differences among proportions, we applied Pearson's chi-square (χ2) test. Furthermore, we used bivariate and multivariate logistic regression analysis to determine the association between each risk factor such as age, sex, body condition score, origin, breed type, and cattle management style with the incidence of the infections. In this analysis, a *p*-value less than 0.05 at the 95 % confidence level was considered statistically significant.

## 3 Results

### 3.1 Characteristics of cattle

In the current study, 384 cattle were included. The highest number of cattle, 140 (36.5 %), were from Abchikili Zuriya *Kebele*. The sampled cattle comprised both local and cross-breed types, with the majority, 288 (75.0 %), being local breeds. Approximately 59.4 % of the cattle in the study were female. In terms of age groups, 144 (37.5 %) and 132 (34.4 %) of cattle were aged 2–5 and over 5 years, respectively. Around 12.5 % and 53.1 % of the cattle were in good and medium body conditions, respectively. The majority of the cattle, 332 (86.5 %), were kept under extensive management systems (Table 1).

**Table 1:** Characteristics of studied cattle south Achefer district, northwest Ethiopia, 2020 (*N* = 384).

Characteristics	Category	Frequency	Percent
Origin of cattle (Kebeles)	Abchikli Zuriya	140	36.5
	Ker Gurach	134	34.9
	Yeboden Gergista	110	28.6
Breed types	Local	288	75.0
	Cross	96	25.0
Sex	Female	228	59.4
	Male	156	40.6
Age (years)	<2	108	28.1
	2–5	144	37.5
	>5	132	34.4
Body condition	Poor	132	34.4
	Medium	204	53.1
	Good	48	12.5
Cattle Management systems	Extensive	332	86.5
	Semi-intensive	36	9.4
	Intensive	16	4.2

### 3.2 Prevalence of bovine Schistosoma infections

Among the 384 faecal samples that were analyzed, the overall prevalence of bovine *Schistosoma* was 9.6 %. Although there were differences in the prevalence of bovine *schistosoma* among the different characteristics of cattle, none of them were observed to be statistically significant. Out of the three peasant associations, cattle from Abichikli Zuriya had the highest prevalence (10.7 %), followed by those from Ker Gurach (10.4 %).

Cattle less than 2 years old were found to have lower rates of *Schistosoma bovis* infection compared to older cattle. The prevalence of bovine *Schistosoma* was lower in cattle with good body condition (6.2 %) compared to those with medium (9.8 %) and poor body condition (10.6 %). However, the difference in proportions was not statistically significant (chi-square (χ2) = 0.781, *p*-value = 0.677) (Table 2).

**Table 2:** Prevalence of bovine *Schistosoma* infection in relation to different cattle characteristics, South Achefer district, Amhara region, northwest Ethiopia, 2020 (*N* = 384).

Characteristics	Category	Bovine *Schistosoma* species infection status		χ2	P-value
		Positive n (%) Negative n (%)			
Origin of cattle (Kebeles)	Abchikli Zuriya	15(10.7)	125 (89.3)	0.994	0.608
	Ker Gurach	14 (10.4)	120 (89.6)		
	Yeboden Gergista	8 (7.3)	102 (92.7)		
	Total	37 (9.6)	347(90.4)		
Breed types	Local	28(9.7)	260(90.3)	0.010	0.920
	Cross	9 (9.4)	87(90.6)		
Sex	Female	21(9.2)	207(90.8)	0.116	0.733
	Male	16 (10.3)	140(89.7)		
Age (years)	<2	7(6.5)	101(93.5)	1.788	0.409
	2–5	15(10.4)	129(89.6)	1.788	0.409
	>5	15(11.4)	117(88.6)		
Body conditions	Poor	14(10.6)	118(89.4)	0.781	0.677
	Medium	20(9.8)	184(90.2)		
	Good	3(6.2)	45,993.8)		
Cattle Management systems	Extensive	33(9.9)	299(90.1)	1.831	0.400
	Semi-intensive	4(11.1)	32(88.9)		
	Intensive	0	16(100)		

In terms of breed types, a similar percentage of bovine *Schistosoma* was found in local breeds (9.4 %) and crossbreed cattle (9.4 %). None of the cattle kept under intensive management tested positive for bovine *Schistosoma* infection. Additionally, the variance in the prevalence of bovine *Schistosoma* infection among cattle under different management systems was not statistically significant (χ2 = 1.8, p-value = 0.400) as shown in Table 2.

### 3.3 Prevalence of Fasciola infections

The study found that 54.2 % (208 out of 384) of the cattle examined were affected by *Fasciola hepatica* (*F. hepatica*), while *Fasciola gigantica* was not detected. There were significant differences in the prevalence of *F. hepatica* based on cattle breed types, age, body condition, and cattle management systems (*P* < 0.05) (Table 3). Specifically, crossbreed cattle showed a higher prevalence of *F. hepatica* (77.1 %) compared to local breeds (46.5 %), and this difference was statistically significant (χ2 = 27.07, *P* < 0.001). Additionally, young cattle under 2 years of age had a significantly higher prevalence of *F. hepatica* (86.1 %) compared to older cattle (χ2 = 63.22, *P* < 0.001) (Table 3).

**Table 3:** Prevalence of *F. hepatica* in relation to some characteristics of cattle, south Achefer distrcit, Amhara region, northwest Ethiopia, 2020 (*N* = 384).

Characteristics	Category	*F. hepatica* status		χ2	P-value
		Positive n (%) Negative n (%)			
Origin of cattle	Abchikli Zuriya	78(55.7)	62(44.3)	0.908	0.613
	Ker Gurach	68(50.7)	66(49.3)		
	Yeboden Gergista	62(56.4)	48(43.6)		
	Total	208(54.2)	176(45.8)		
Breed types	Local	134(46.5)	154(53.5)	27.077	<0.001
	Cross	22(77.1)	32(22.9)		
Sex	Female	131(57.5)	97(42.5)	2.446	0.118
	Male	77(49.4)	79(50.6)		
Age (Years)	<2	93(86.1)	15(13.9)	63.224	<0.001
	2–5	55((38.2)	89(61.8)		
	>5	60(45.5)	72(54.5)		
Body condition	Poor	86(65.2)	46(34.8)	25.302	<0.001
	Medium	111(54.4)	93(45.6)		
	Good	11(22.9)	37(77.1)		
Cattle Management systems	Extensive	199(59.9)	133(40.1)	33.464	<0.001
	Semi-intensive	5(13.9)	31(86.1)		
	Intensive	4(25.0)	12(75.0)		

χ2 = chi-square, N = total number of cattle included in the study.

A statistically significant higher prevalence (65.2 %) of *F. hepatica* was observed in cattle with poor body conditions than the others (χ2 = 25.302, *P* = 0.00). About the cattle management system, the prevalence of *F. hepatica* was highest in extensively managed cattle (59.9 %) compared with semi-intensively (13.9 %) and intensively (25.0 %) managed ones, and the difference was statistically significant (χ2 = 33.46, P < 0.001). Although there were variations in the proportions of *F. hepatica* infections among categories of cattle origin and age, the differences were not statistically significant (Table 3).

### 3.4 Logistic regression analysis for *F. hepatica* with associated cattle characteristics

Both univariate and multivariate logistic regression analyses were used to determine the strength of the association between the occurrence of *F. hepatica* and its risk factors. The multivariate logistic regression analysis showed that breed type, age, and body condition were significantly associated factors with the occurrence of *F. hepatica* in the study area. The odds of being infected by *F. hepatica* were about two times higher in cross-breed cattle than in local ones, and the association was statistically significant (AOR: 1.88, 95 % CI: 1.03–3.44, *P* = 0.041).

In the present study, the age of cattle was also observed to be significantly associated with the occurrence of *F. hepatica* infection. Cattle less than two years old were observed to be more than seven times more likely to contract the helminth than those above five years old (AOR: 7.31, 95 % CI: 3.54–15.08, *P* < 0.001). Concerning cattle body condition, those with poor body condition were observed to be infected by *F. hepatica* more than four times as compared to cattle with good body condition, and this association was statistically significant (AOR: 4.85, 95 % CI: 1.94–12.14, P < 0.001) (Table 4).

**Table 4:** Bivariate and multivariate logistic regression analysis of *F. hepatica* with the associated risk factors among cattle in South Achefer district, northwest Ethiopia, 2020.

Characteristics	Category	*F. hepatica status*		COR (95 % CI)	P. value	AOR (95 % CI)	P. value
		Pos (%) Neg (%)					
Origin of cattle	AZ	78(55.7)	62(44.3)	1			
	KG	68(50.7)	66(49.3)	0.82(0.51,1.32)	0.410	NA	
	YG	62(56.4)	48(43.6)	1.03(0.62,1.70)	0.918	NA	
Breed types	Local	134(46.5)	154(53.5)	1		1	
	Cross	22(77.1)	32(22.9)	3.87(2.28,6.56)	<0.001	1.88(1.03,3.44)	0.041
Sex	Female	131(57.5)	92(42.5)	1.39(0.92,2.09)	0.118	1.35(0.79,2.33)	0.275
	Male	77(49.4)	79(50.6)	1		1	
Age (years)	<2	93(86.1)	15(13.9)	7.44(3.91,14.17)	<0.001	7.31(3.54,15.08)	<0.001
	2–5	55((38.2)	89(61.8)	0.74(0.46,1.20)	0.222	0.68(0.37,1.24)	0.209
	>5	60(45.5)	72(54.5)	1		1	
Body condition	Poor	86(65.2)	45(34.8)	6.29(2.93,13.48)	<0.001	4.85(1.94,12.14)	<0.001
	Medium	111(54.4)	93(45.6)	4.012(1.94,8.31)	<0.001	1.57(0.67,3.70)	0.297
	Good	11(22.9)	37(77.1)	1		1	
Cattle Management systems	Extensive	199(59.9)	133(40.1)	4.49(1.42,14.21)	<0.001	1.39 (0.41, 4.72)	0.596
	Semi-intensive	5(13.9)	31(86.1)	0.48(0.111,2.113)	0.3334	0.45(0.09,2.27)	0.335
	Intensive	4(25.0)	12(75.0)	1		1	

AZ: Abchikli Zuriya, KG; Ker Gurach, YG: Yeboden Gergista COR = Crude odds ratio, CI = Confidence interval, AOR = Adjusted odds ratio, NA = Not applicable.

## 4 Discussion

The study found that the overall prevalence of bovine *Schistosoma* species infections was 9.6 % (*n* = 37), and *F. hepatica* infections were 54.2 % (*n* = 208). The higher prevalence of *F. hepatica* infections compared to *Schistosoma* species may be due to the extensive cattle management system, local breed type, and the amphibious nature of *F. hepatica*. The metacercaria can survive up to 3 months after harvesting hay from the endemic highland area, which is consumed by cattle in arid and lowland areas, particularly during the dry season. The prevalence of bovine *Schistosoma* species in this study is almost comparable to previous studies in different parts of Ethiopia, such as in Fogera District (10.17 %, 10.3 %) (Mengistu et al., 2012; Belete and Engdaw, 2015) and Dangla District (11.5 %) (Alemayehu and Asrat, 2015).

However, the prevalence of bovine *Schistosoma* species in this study is lower than in other studies conducted in Bahir Dar areas (16.5 %, 24.6 %) (Aragaw and Tilahun, 2019; Lulie and Guadu, 2014). The higher prevalence of bovine *Schistosoma* species in the above studies might be due to the availability of suitable snail hosts because of Lake Tana and other wetlands in the area, which in turn increases the transmission of the disease. One possible explanation for the decreasing prevalence of bovine *Schistosoma* species in the current study could be the growing practice of keeping cattle indoors in the study area in recent years.

The prevalence of *F. hepatica* in this study was comparable with the previous study conducted in Kiyu District of eastern Shoa (54.2 %) (Mohammed et al., 2018), but lower compared to studies conducted in Dangilla, 90.6 % (Asmare and Samuel, 2015) and in south Gondar 83.1 % (Mulualem, 1998), but somehow greater than studies conducted in Zenzelma around Bahir Dar 27.0 % (Legesse et al., 2017) and Horro district of Kellem Wollega Zone 27.6 % (Tulu and Gebeyehu, 2018). The lower prevalence of *F. hepatica* in the current study area, compared to previous studies, may be due to the drying of water bodies, stagnant water areas, and the expansion of farmland in the present study area. These changes limit the transmission of the infection.

Although the association was not statistically significant (*P* > 0.05), there was a slight variation in the prevalence of bovine *Schistosoma* infections across the breeding types of the study cattle. In the case of *F. hepatica* infection, cross-breeds have shown a higher chance of being infected than the local breeds. The above association was also statistically significant (*p* < 0.05). In support of our finding, a higher prevalence (25.8 %) of *F. hepatica* was reported in cross-breed cattle than in local ones (16.7 %) in and around Bahir Dar (Woldemaryam, 2008). This difference might be because of the local breed's better adaptation and immunity compared to pure or cross-breeds if there is equal exposure to the parasite in question. On the contrary, a higher prevalence of *F. hepatica* in local breeds than in cross-breed cattle was previously reported in the Dangla district (Alemayehu and Asrat, 2015). Moreover, a similar study also reported a higher prevalence in the local breed than in the cross-breed in Gondar (Gebre, 2010). This might be due to the lower exposure of cross-breed cattle to the marsh areas compared with local cattle breeds.

In this study, a lower prevalence of bovine *Schistosoma* and *Hepatica* infections was reported in male cattle compared to female cattle. However, there was no statistically significant difference between the two sexes. This aligns with a study in the Dangla district, which reported a slightly higher prevalence in female cattle (Alemayehu and Asrat, 2015). The results indicated that both sexes were at the same risk of acquiring the infections because of similar exposure to the risk factors, as there were no restrictions on movement for grazing or contact with the parasites in terms of sex. In addition, since both sex groups were grazing on similar contaminated pasture land and had access to identical water points, they were more susceptible to the risk of contracting the infection. However, the difference in the previous report of a relatively higher figure in females than in males might be due to the unproportional representation of male and female cattle in the study.

The study showed that young cattle (under two years old) had a much higher prevalence of *F. hepatica* compared to older cattle. This higher prevalence in younger cattle may be due to their lower immunity, making them more susceptible to infection. In contrast, older cattle showed a reduced prevalence of *F. hepatica* likely due to the development of acquired resistance against the parasites. This resistance may limit the worms' ability to reproduce and facilitate the release of parasitic eggs in the faeces. These findings align with a previous study conducted in Fogera and Bahir Dar (Mengistu et al., 2012; Lulie and Guadu, 2014).

The statistical analysis of this study showed that the body condition score (BCS) had a significant influence on the prevalence of *F. hepatica* in the study area. The highest prevalence was recorded in cattle with poor body condition, which are more than four times the odds of being infected. The cause may be connected to the body's defence mechanism in cattle, which states that healthier animals have stronger immune systems than unhealthy animals (Sordillo, 2016). As a result, animals with lower body condition scores were more likely to become infected than other cattle groups. Similarly, a prior study confirmed that animals with low body condition scores have higher infection rates (Merawe et al., 2014). However, immunity was predominantly mediated by worm fecundity, as previously mentioned, rather than acting to completely prevent infection (Lawerence, 1979). Another possible explanation is that farmers feed their weaker animals green fodder cultivated in marshy places to compensate for their BSC.

The grazing habits in extensive cattle management systems may lead to a potential infection of *F. hepatica* due to the frequent contact of animals with water bodies. In a semi-intensive management system, cattle are kept indoors and partly outdoors, which might increase the risk of acquiring the infection while grazing. However, animals kept in an indoor intensive management system are provided with adequate food and clean water, which positively supports cattle health. The current report agreed with a study conducted in Addis Ababa (Dereje and Gebrehiwot, 2018) in which *F. hepatica* was lower in intensive management systems than in semi-intensive and extensive management systems. The primary explanation may be attributed to the management approach, which keeps the animals indoors where there is less chance of disease contact than in semi-intensive and extensive systems. They could come into contact with the illness if green feed, collected from marshy areas where the parasite's metacercarial stage is present, is added to feeding and watering troughs.

In conclusion, the present study revealed that both bovine *Schistosoma* and *Fasciola* infections remain the major diseases, resulting in considerable public health and economic burdens in the study area. The high prevalence of bovine *Schistosoma* and *Fasciola* infections in the present study area might have been influenced by the water supply from a small irrigation programme in the area, the presence of the Blue Nile River, and the formation of large swampy areas, ponds, and stagnant water bodies. The outdoor grazing practice of cattle in the study area was also responsible for the higher prevalence of bovine *Schistosoma* and *Fasciola* infection. Faces excreted outside easily contaminate the water source that cattle use for drinking. The presence of suitable habitats for the development and multiplication of intermediate hosts aggravated *S. bovis* and *F. hepatica* infections in cattle. Hence, appropriate and effective disease and snail control strategies should be implemented in the study area.

## Ethical considerations

The protection of the study's livestock was conducted in accordance with national and international norms. The College of Natural and Computational Science (CNCS), University of Gondar's ethical committee approved the study (CNCS/10/638–25-5-2020). Informed consent was obtained from each cattle owner after they were informed about the purpose of the study. Treatment was provided at no cost for animals infected with *S. bovis* and *F. hepatica*, in accordance with recommendations and in coordination with veterinary professionals.

## Funding

No funding was obtained for this study.

## Authors' contribution

ST: Conceptualized the study, collected and analyzed the data, developed methodology, supervised the study, participated in the draft and final write-up of the manuscript.

MY: Conceptualized the study, developed methodology, validated study, supervised the study, analyzed data, and wrote the original draft of the study.

AA: Developed methodology, supervised the study, analyzed data, and wrote the original draft of the study.

TY: Developed methodology, supervised the study, analyzed data, and wrote the original draft of the study.

## Declaration of competing interest

The authors declare that they have no known competing financial interests or personal relationships that could have appeared to influence the work reported in this paper.

## Data Availability

The corresponding authors can share data when reasonable requests emerge.
